# Mechanisims of asthma and allergic disease – 1075. Probiotics in infants for prevention of allergic disease and food hypersensitivity

**DOI:** 10.1186/1939-4551-6-S1-P72

**Published:** 2013-04-23

**Authors:** John Sinn

**Affiliations:** 1 Obstetrics, Gynaecology and Neonatology, University of Sydney, Sydney, Australia

## Background

Probiotic (‘healthy’ bacteria) added to infant feeds have the potential to prevent sensitisation of infants to dietary allergens.

## Methods

Standard methods of the Cochrane Neonatal Review Group were used. Searches were updated October 2011. Randomised and quasi-randomised controlled trials that compared a probiotic to control; or probiotic with added prebiotic (synbiotic) to control were eligible.

## Results

Sixteen studies were eligible. Several ongoing studies and completed studies with no reported allergy results were identified. Eight studies reported adequate randomisation and allocation procedures and used a placebo. Only one study reported <10% losses after allocation. Overall, meta-analysis of 12 studies found a significant reduction in infant eczema (1876 infants; RR 0.77; 95% CI 0.67, 0.89; RD -0.07; -0.12, -0.03; NNT 14; 8, 33] from use of probiotic. Moderate (I^2^=32%) heterogeneity between studies was found. In subgroup analysis, a significant reduction in eczema was found in infants selected for risk of allergy and unselected infants; infants predominately breast fed; infants treated with L. Rhamnosus (3 studies, 542 infants, RR 0.61; 0.45, 0.82); infants treated with a combination probiotic B. bifidum, B. lactis and L. acidophilus (1 study, 68 infants; RR 0.58; 0.34, 0.97); and infants given a synbiotic (1 study, 925 infants; RR 0.81; 0.66, 0.99). There was no significant difference in all allergy, asthma, atopic eczema, allergic rhinitis, or food hypersensitivity.

## Conclusions

Further evidence is required before a probiotic or synbiotic can be recommended for prevention of allergy. A well powered, independent trial is required to answer this question.

